# Malignant mesenchymal tumor with leiomyosarcoma, rhabdomyosarcoma, chondrosarcoma, and osteosarcoma differentiation: case report and literature review

**DOI:** 10.1186/1746-1596-6-35

**Published:** 2011-04-15

**Authors:** Yao-Feng Li, Cheng-Ping Yu, Seng-Tang Wu, Ming-Shen Dai, Herng-Sheng Lee

**Affiliations:** 1 Department of Pathology, Tri-Service General Hospital, Taipei, Taiwan, China; 2 Division of Urology, Department of Surgery, Tri-Service General Hospital, Taipei, Taiwan, China; 3 Division of Oncology, Department of Internal medicine, Tri-Service General Hospital, Taipei, Taiwan, China; 4 National Defense Medical Center, Taipei, Taiwan, China

## Abstract

A case of malignant mesenchymoma of the bladder containing leiomyosarcoma, rhabdomyosarcoma, chondrosarcoma, osteosarcoma, and myxomatous components is described. The primary pedunculated tumor measuring 14 × 13 × 7 cm and weighing 343 g arose from the left trigone of the bladder and was treated by total cystectomy. The histogenesis of malignant mesenchymomas and their optimal management strategy and prognosis remain uncertain. Herein, we present the fifth case of malignant mesenchymoma of the urinary bladder to be reported in the literature, which presented five unrelated differentiated tissues more than did previously reported cases.

## Background

Malignant mesenchymoma, which was described by Stout in 1948, is defined as a malignant soft tissue tumor that consists of two or more distinctly different mesenchymal components in addition to fibrosarcomatous elements. A review of the literature revealed only four cases of bladder sarcomas that fit the criteria.

## Case presentation

A 77-year-old Taiwanese man had a multiyear history of symptoms of micturition difficulty. Gross hematuria with severe pain had developed suddenly in the previous month. The patient is a retired military soldier had neither history of cigarette smoking nor habbit of alcohol comsumption. He had a history of hypertension and type II diabetes mellitus for 20 years controlled with regular medication. He didn't exposures to paint components or eat undercooked meat. There is no specific illness could be traced from the patient's family pedigree. On physical examination, the patient appeared to be recently poorly nourished and had normal vital signs and mild elevated blood pressure at 151/88 mmHg. His lungs were clear, heart sounds were normal, and the abdomen was soft, with no masses and no tenderness. There was no edema of the legs. In laboratory data, the results of a complete blood count and the levels of electrolytes were normal. However, serum total protein, albumin, creatinine (Cr), urea nitrogen (BUN), and glucose were abnormal and showing hypoproteinemia (5.5 g/dL), hypoalbuminemia (3.0 g/dL), abnormal renal function with moderate azotemia (BUN/Cr = 36/1.3), and hyperglycemia (179 g/dL).

Real-time sonographic evaluation of the abdomen showed increased echogenicity of bilateral kidneys. The sizes of the kidneys were 9 cm on the right and 10.3 cm on the left. Mild dilatation of left renal pelvis is also noted. Furthermore, one lobulated mass with urinary bladder wall thickening was also identified.

**Figure 1:**
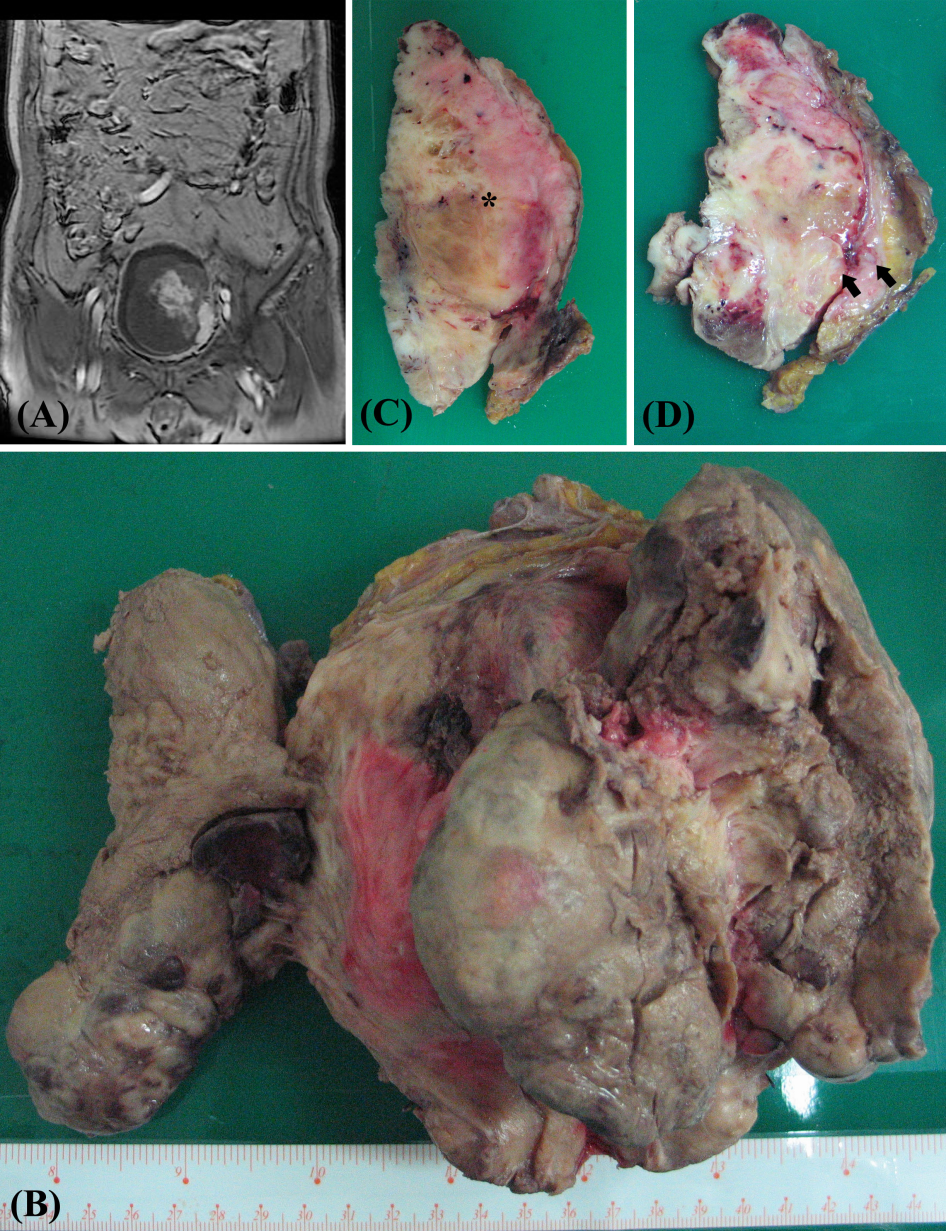
**(A) Magnetic resonance imaging (MRI) of the pelvis demonstrated a large mass in the urinary bladder that shows focal contrast medium enhancement on the left side with adjacent vesical wall thickening and obliteration of the lower signal intensity on T2WI**. (B) Macroscopically, the urinary bladder contained a nonencapsulated, huge, and lobular mass, which was located in the left trigone and measured 14 × 13 × 7 cm in size. (C) and (D) On the cut surface, this tumor was composed of a predominant white, myofibrous-like component, and myxomatous (asterisk), hemorrhage, and tumor necrosis foci. Several hard osteochondroid-like nodules were also palpable within this tumor (arrow).

Magnetic resonance imaging (MRI) of the pelvis demonstrated a large mass in the urinary bladder. The mass shows focal contrast medium enhancement on the left side with adjacent vesical wall thickening and obliteration of the lower signal intensity on T2WI (Figure 1A). Urinary bladder malignancy was suspected, and the differential diagnoses included urothelial cell carcinoma with blood clot, sarcoma, or other benign neoplastic mass.

Transurethral resection of the urinary bladder tumor was performed. Pathological examination led to a diagnosis of high-grade sarcoma composed of myofibrous differentiation, focal myxomatous pattern, and a few chondroid changes with marked nuclear atypia and increased mitotic figures (more than 10/10 high power field). Radical cystectomy was performed. The patient currently remains well and prepares to undergo adjuvant chemotherapy.

## Results

Macroscopically, the urinary bladder contained a nonencapsulated, huge, and lobular mass, which was located in the left trigone and measured 14 × 13 × 7 cm. On its cut surface, this tumor was composed of a predominant white, myofibrous-like component (70%), with myxomatous (20%), hemorrhage, and tumor necrosis (7%) foci. Several hard osteochondroid-like nodules were also palpable, and they measured around 3% of the whole tumor lesion (Figure 1B, C, and 1D).

**Figure 2:**
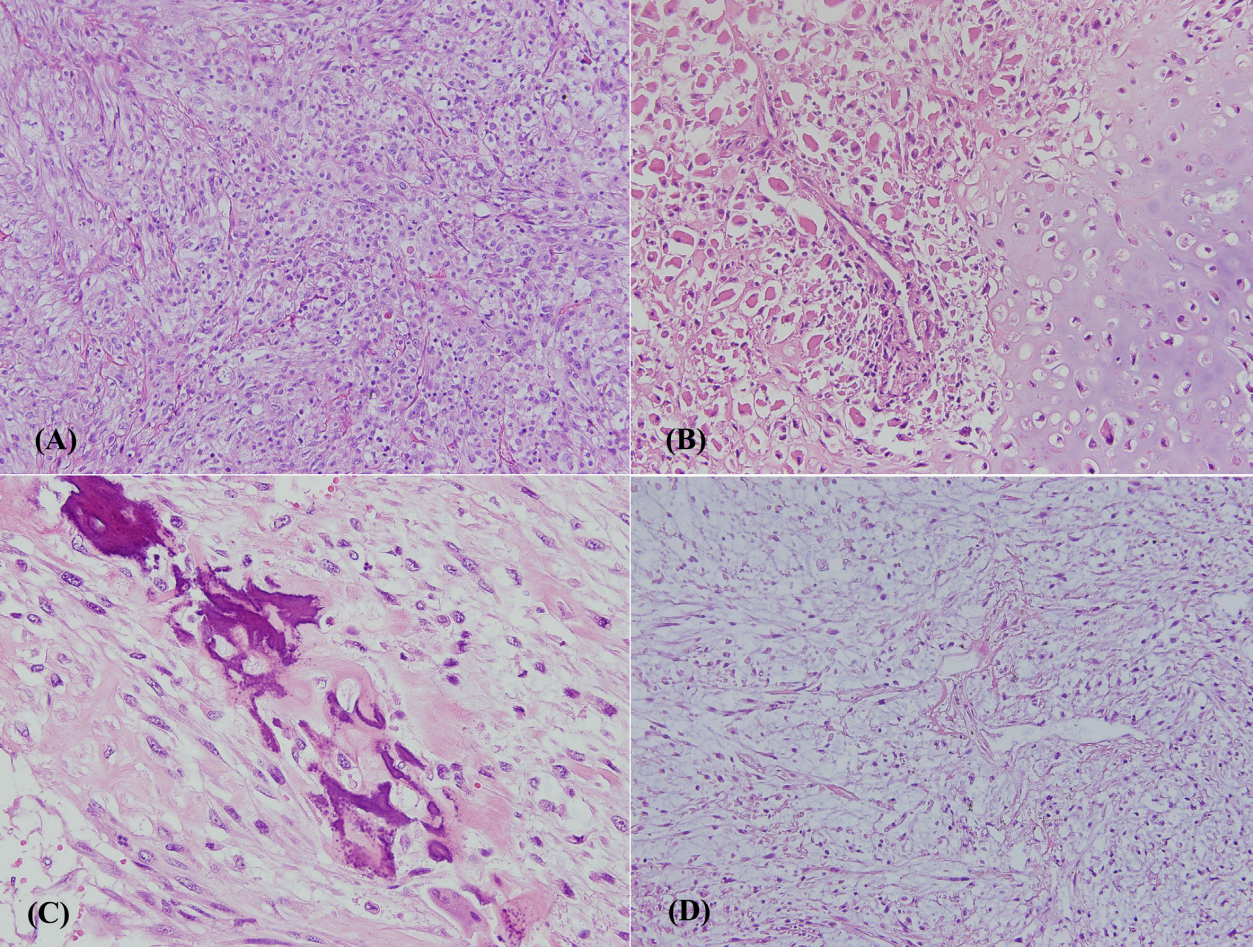
**Microscopically, the tumor cells exhibited pleomorphic spindle tumor cells composed of (A) predominant myofibrous differentiation, (B) rhabdoid with chondroid differentiation, (C) osteoid differentiation**. Unlike metaplasia, neoplastic osteosarcoma cells are usually surrounded by deposited osteoid material and (D) focal myxomatous differentiation (hematoxylin and eosin staining, ×200).

**Figure 3:**
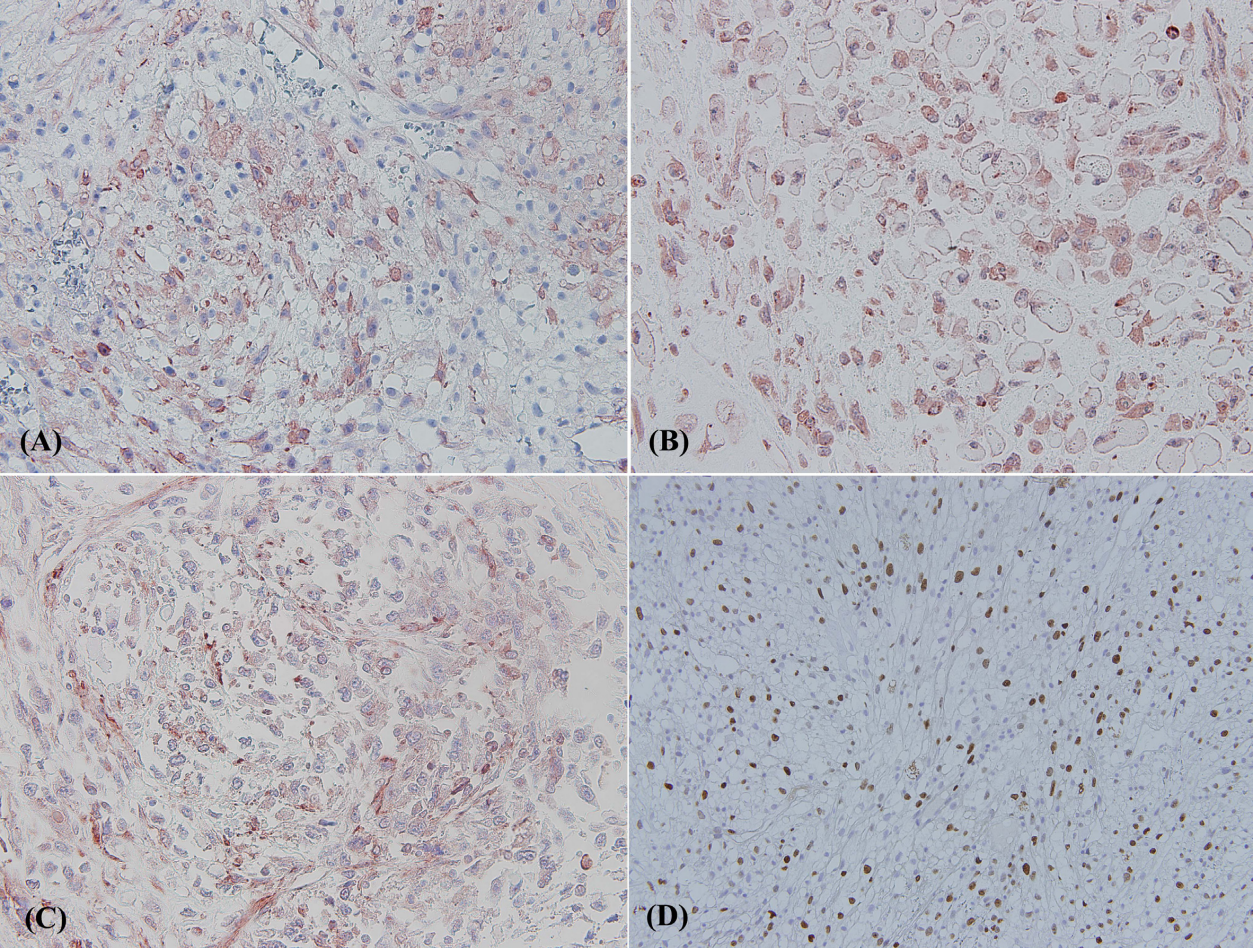
**This high-grade sarcoma shows muscle differentiation because of focally positive immunoreactivity to (A) calponin, (B) myoglobulin, and (C) smooth muscle actin antibodies**. (D) It also expressed a high proliferation index by Ki-67 staining (×200). To view the virtual glass slides for this figure please see here http://diagnosticpathology.slidepath.com/webViewer.php?snapshotId=1304063671 and http://diagnosticpathology.slidepath.com/webViewer.php?snapshotId=1304063705.

Microscopically, the tumor cells exhibited pleomorphic spindle tumor cells composed of predominant myofibrous differentiation with focal rhabdoid, chondroid, osteoid, and focal myxomatous differentiation. Nuclear atypia, focal clear cytoplasm, marked increased mitotic figures, focal hemorrhage, and tumor necrosis are also noted (Figure 2). The spindle tumor cells showed focally positive immunoreactivity to smooth muscle actin, calponin, and myoglobulin antibodies (Figure 3A, B, and S3C). This tumor also shows very high proliferative activity through Ki-67 immunohistochemical staining (Figure 3D). Immunohistochemical findings are summarized in Table 1. No epithelial component was seen. The surgical margins were all free of tumor cells. Lymphovascular space invasion was not identified. According to the clinical information, histopathological features, and immunoprofiles, it was a case of malignant mesenchymoma, and the AJCC sarcoma staging was pT2bN0M0, stage III.

**Table 1:** Immunohistochemical results for all kinds differentiated components of malignant mesenchymoma

Antibody	Clone	Dilution	leiomyosarcoma	rhabdomyosarcoma	chondrosarcoma	osteosarcoma
**Cytokeratin-7**	OV-TL12/30	1:500	*Negative*	*Negative*	*Negative*	*Negative*
**Cytokeratin-20**	Ks 20.8	1:100	*Negative*	*Negative*	*Negative*	*Negative*
**Vimentin**	clone V9	1:200	***Positive***	***Positive***	***Positive***	***Positive***
**CD10**	56C6	1:50	***Positive***	*Negative*	*Negative*	*Negative*
**CD34**	QBEnd-10	1:100	*Negative*	*Negative*	*Negative*	*Negative*
SMA*	HHF35	1:75	***Positive***	***Positive***	*Negative*	*Negative*
**Calponin**	CALP	1:50	***Positive***	***Positive***	*Negative*	*Negative*
**Caldesmon**	h-CD	1:100	*Negative*	*Negative*	*Negative*	*Negative*
**Myoglobulin**	Lot 127	1:400	***Positive***	***Positive***	*Negative*	*Negative*
**Myo-D1**	5.2F	1:100	*Negative*	*Negative*	*Negative*	*Negative*
**Myogenin**	F5D	1:100	*Negative*	*Negative*	*Negative*	*Negative*
**S100**	4C4.9	1:100	*Negative*	*Negative*	*Negative*	*Negative*
**HMB-45**	M0634	1:50	*Negative*	*Negative*	*Negative*	*Negative*

*SMA, smooth muscle actin

## Discussion

Primary sarcomas of the urinary bladder are uncommon and mostly originate from muscle such as rhabdomyosarcoma, which is dominant in children, or leiomyosarcoma, which is dominant in adults. Other rare sarcomas documented in the literature include primary osteosarcoma (31 cases) [1], malignant fibrous histiocytoma (30 cases) [2], primary angiosarcoma (15 cases) [3], and several cases of malignant mesenchymoma.

Malignant mesenchymoma is the rarest sarcoma of the urinary bladder, and it was defined by Stout as a sarcoma comprising two or more unrelated differentiated tissue elements other than a fibrosarcoma component. Our case presents five unrelated differentiated tissues other than fibrosarcoma. To our knowledge, in the English-language medical literature, only four cases of malignant mesenchymoma of the urinary bladder have been previously reported [4-7]. The major clinical and pathological features of these cases and our case are summarized in Table 2. The available data for malignant mesenchymoma of the urinary bladder show that it predominantly occurs in male patients who are older than 40 years. The highest incidence is in the eighth decade. The size of the tumor is often more than 10 cm, and an advanced AJCC sarcoma stage is common when diagnosed.

**Table 2:** Major clinical and morphologic features of reported malignant mesenchymal tumor of urinary bladder

	Age/Sex	Size of tumor	Histology of sarcomatoid component	AJCC stage of sarcoma	Management	Clinical behavior
Case 1 [4]	83/M	NA	Reported as cancerous mixed tumors	NA	NA	NA
Case 2 [5]	44/F	NA	Reported as osteogenic leiomyosarcoma	NA	NA	NA
Case 3 [7]	80/M	18 × 10 × 9 cm	Malignant pleomorphic spindle cells with leiomyomatous, chondromatous, osteoid and myxomatous differentiation	T2bN0M0, Stage III	Refused total cystectomy and received chemotherapy and radiotherapy	Died 22 months after diagnosis
Case 4 [6]	72/M	NA	Predominantly a leiomyosarcomatous component with foci of malignant bone, cartilage and myxomatous areas.	NA	NA	NA
Present case	78/M	14 × 13 × 7 cm	Malignant pleomorphic spindle cells with leiomyomatous, rhabdoid chondromatous, osteoid and myxomatous differentiation	T2bN0M0, Stage III	Receive total cystectomy	Alive 2 month after diagnosis

Abbreviation: NA, not available.

Malignant mesenchymoma can occur at all locations in the body, including the retroperitoneum, soft tissue of the lower limbs, heart [8], mediastinum [9], pleura [10], liver [11], orbit [12], bone [13], larynx [14], thyroid [15], testis [16], uterine [17], and urinary bladder [4-7]. Such tumors more frequently develop in the retroperitoneum and the soft tissue of the lower limbs.

Several neoplasms remain that could qualify as malignant mesenchymoma according to the definition, but these are frequently treated as distinct and separate entities, such as malignant triton tumor, ectomesenchymoma, dedifferentiated liposarcoma, and chondrosarcoma with a second differentiated component [18].

Malignant mesenchymoma appears to arise from a primitive mesenchymal cell with the capacity for totipotent differentiation, but the histogenesis remains uncertain.

One should be careful when diagnosing malignant mesenchymoma, because some sarcomas are easily combined with bone and chondroid metaplasia that could mimic this diagnosis. The histological distinction between neoplastic and metaplastic bone is based on the pattern of the deposited bone, the cytological features of the bone-forming cells, and the cellular composition of the intratrabecular tissue. Metaplastic bone often has a lamellar architecture, and it is usually organized around areas of hemorrhage or portions of tumor. In contrast, the neoplastic osteosarcoma is usually surrounded by deposited osteoid material (Figure 2C).

The prognosis of malignant mesenchymomas remains controversial. Malignant mesenchymomas are commonly accepted as high-grade malignant neoplasms. Bradythe had reported two- and three-year survival rates of 75% and 37%, respectively, in eight femoral and retroperitoneal cases [19]. However, Newman and Fletcher suggested low-grade malignant behavior for malignant mesenchymoma bases in six cases, four of which had less than five years of follow-up [20]. Adachi reported that a patient aged under 40 years and the presence of a rhabdomyosarcomatous component correspond to a poor prognosis, but there is no significant prognosis difference with respect to gender, tumor site, tumor size, or MIB-1-labeling index [21].

Because of its extreme rarity, there are insufficient data to suggest the best modality or combination of treatments for this condition [11,22,23]. Chemotherapy and radiotherapy were ineffective for the soft parts of the sarcoma, including the malignant mesenchymoma, but the efficacy of chemotherapy with doxorubicin plus ifosfamide and cyclophosphamide, vincristine, doxorubicin, and dacarbazine has recently been reported. Thus, a multidisciplinary approach including surgery, radiotherapy, and chemotherapy may be useful for these tumors [23].

## Conclusions

In summary, malignant mesenchymoma is a rare tumor, and this is the fifth reported case of a malignant mesenchymoma of the urinary bladder. Because of the limited experience with this extremely rare tumor, there are insufficient data to suggest the optimal management strategy and prognosis for malignant mesenchymoma of the urinary bladder.

## Competing interests

The authors declare that they have no competing interests.

## Authors' contributions

YFL drafted the manuscript. CPY carried out the immunohistochemical stains evaluation. STW and MSD provide clinical information. HSL supervised this manuscript. All authors read and approved the final manuscript.
